# H1N76/77 deamidation facilitates chromatin remodelling and genome stability during DNA damage repair

**DOI:** 10.1002/ctm2.70440

**Published:** 2025-08-15

**Authors:** Tingting Feng, Mengyan Li, Chenmiao Hu, Yuan Tian, Wei‐Guo Zhu

**Affiliations:** ^1^ Department of Biochemistry and Molecular Biology International Cancer Centre Guangdong Key Laboratory of Genome Instability and Human Disease Prevention Marshall Laboratory of Biomedical Engineering Shenzhen University Medical School Shenzhen China

**Keywords:** deamidation, DNA damage repair, linker histone H1

## Abstract

Chromatin relaxation is a permissiven progress for DNA repair through enabling repair factors to access the damaged DNA. Linker histone H1 is important in maintaining chromatin compaction under physiological state. The recent evidence highlights the importance of H1 modifications in response to cellular stress. Following DNA double‐strand breaks, the metabolic enzyme phosphorylated CTP synthase 1 (CTPS1) functions as a deamidase, catalyzing the rapid conversion of H1 residues Asn76 and Asn77 into aspartate. This modification enables subsequent acetylation at Lys75 by the histone acetyltransferase p300, thereby reducing H1‐DNA affinity and promoting chromatin decompaction. This sequential modification‐H1 deamidation followed by acetylation‐facilitates the recruitment of repair factors involving both homologous recombination and non‐homologous end joining repair pathways, and consequently promoting DNA repair. Importantly, high expression of CTPS1 is associated with resistance to radiotherapy in mouse models and clinical cancer patients, suggesting that the CTPS1 may serve as a potential therapeutic target. While targeting CTPS1 may offer opportunities to enhance radiosensitivity of cancer patients, challenges related to specificity and off‐target effects require further studies. This article highlights an emerging role of H1 modification in the DNA damage repair and discusses the therapeutic potential of manipulating H1 deamidation in cancer treatment.

## 1 HISTONE H1 AND CHROMATIN RELAXATION DURING DNA DAMAGE REPAIR

DNA in eukaryotic cells is continually being damaged in many ways; among those, double‐strand break (DSB) is the most severe type of damage because of the induction of genomic instability. To preserve genomic integrity, mammalian cells repair DSBs mainly by non‐homologous end joining (NHEJ) and homologous recombination (HR) pathways. After DNA damage, damaged chromatin first becomes more accessible to recruit DNA repair factors to DNA damage sites, ensuring an efficient DNA damage repair.^1^, ^2^ Increasing reports have provided mechanisms which enable accessibility to damaged chromatin through chromatin remodelling factors and histone‐modifying enzymes.^3^ One of the best characterised changes in chromatin organisation is associated with increased acetylation of histones H2A and H4 on nucleosomes at DSBs.^4^, ^5^, ^6^, ^7^ The sensitivity of DNA to nuclease digestion increases after DNA damage,^8^ indicating that linker DNA between nucleosomes is more accessible. Linker histone H1, binding with linker DNA, facilitates the folding of chromatin into a more compact form and functions mainly in chromatin dynamics and gene expression.^9^ While core histones have been extensively studied, our knowledge of H1 modifications and functions was very limited. It has been shown that citrullination of Arginine 54 within the globular domain of the linker histone variant H1.2 is important for its removal from chromatin, leading to decondensation of chromatin during reprogramming to pluripotency.^10^ Lys34 acetylation of H1.4 participates in epigenetic regulation by affecting DNA methylation at specific loci.^11^ Acetylation of H1.4 at Lys26 is related to the formation of facultative heterochromatin.^12^ Recently, some spectrometrical analysis revealed intensive post‐translational modifications (PTMs), including phosphorylation, acetylation, methylation, ubiquitination, and ADP ribosylation,^13^ indicate that these modifications might contribute to chromatin structure and dynamics.^10^ However, the biological functions of these PTMs on chromatin remodelling are poorly understood.

In this study, we first investigated the PTMs of H1 during the early stage of DNA damage repair. We found that deamidation of H1 asparagine 76 and 77 simultaneous deamidation was the most changed modification upon DNA damage. Furthermore, H1 deamidation led to increased H1 nucleosome compaction by upregulating the nearby residue K75 acetylation. Mechanically, H1N76/77 deamidation facilitates the recruitment of histone acetyltransferase p300 that catalyses K75 acetylation, which reduces the binding between H1 and linker DNA, and therefore promotes chromatin relaxation. Altogether, this study uncovers a novel mechanism in which deamidation of H1N76/77 within the globular domain orchestrates histone acetylation and regulates chromatin dynamics during DNA damage repair. Notably, H1N76/77 deamidation induced local conformational changes of H1 around DNA damage sites, which enable p300 recruitment. Conversely, histone acetylation at H1K75 had no effect on deamidation of H1N76/77, reflecting that H1 deamidation is a prerequisite modification for histone further acetylation. We therefore proposed the sequential modifications of deamidation and acetylation in controlling DNA damage‐induced chromatin dynamics.^14^

## 2 PROTEIN DEAMIDATION AND DNA DAMAGE REPAIR

Deamidation is traditionally recognised as a category of spontaneous and irreversible PTMs.^15^ Therefore, deamidation is often associated with protein stability and ageing‐related disease. Converting Asn and Gln into Asp/IsoAsp and Glu/IsoGlu not only alters the composition, but also the structure of proteins that are potentially more “degraded” because their structure is more altered.^15^ In the recent decade, glutamine amidotransferases (GATs)‐mediated deamidation has been reported to occur in key signalling molecules, such as those involved in innate immune defence, to modulate fundamental biological processes.^16^ GATs constitute a family of metabolic enzymes that extract nitrogen from glutamine to synthesise nucleotides, amino acids, glycoproteins, and the enzyme cofactor nicotinamide adenine dinucleotide, which are building blocks for cell growth and proliferation.^17^ Discovering the metabolic‐independent role of GATs expands their role in maintaining cellular homeostasis in response to stress.^18^

In this study, we screened human 11 GATs and revealed that this deamidation is induced by CTP synthase 1 (CTPS1). Mechanically, CTPS1 was phosphorylated and recruited to DSB sites and then catalysed H1 deamidation in an enzymatic activity‐dependent manner. CTPS1 deficiency significantly impaired DNA repair efficiency. Our data uncovered a previously unrecognised role of CTPS1 in modulating H1 PTM and promoting DNA damage repair, positioning CTPS1 at the convergence of chromatin remodelling and DNA repair. Our recent study added evidence of the gain‐of‐function of deamidation, that CTPS1‐catalysed H1 deamidation facilitates chromatin remodelling in response to DNA damage, and lays a foundation for later efficient repair.

## 3 CLINICAL CHALLENGES AND FUTURE PERSPECTIVES

DSBs account for the majority of the cytotoxicity of genotoxic therapies for cancer patients, such as radiation and DNA damage‐based chemotherapeutics.^19^ The efficacy of these therapies is determined by the repair efficiency of these lethal lesions in response to genotoxic stress.^20^ Cancer cells that are resistant to radiotherapy always exhibit high DNA damage repair effectiveness.^21^ Although extensive work has uncovered the molecular mechanisms of radiotherapy resistance, few have been successfully translated into clinical use. Therefore, there is a pressing need to identify key regulators and mechanisms critical to DNA damage repair, to establish a reliable method for predicting and overcoming radioresistance.

**FIGURE 1:**
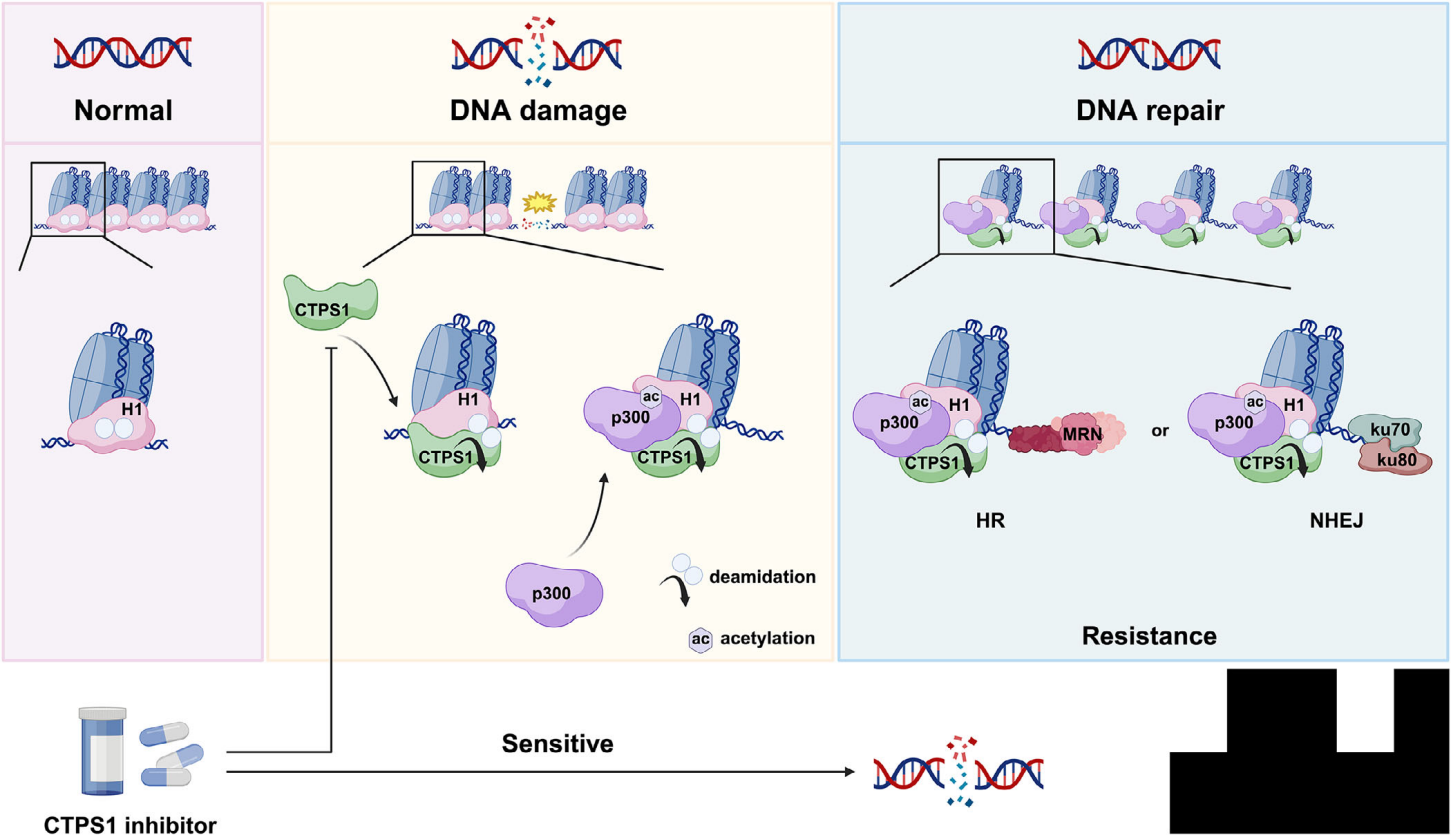
Inhibition of CTP synthase 1 (CTPS1) enhances cellular sensitivity to DNA damage. Under normal conditions, chromatin remains compact with unmodified histone H1. Upon DNA damage, CTPS1 deamidates H1 at Asn76/77, enabling acetylation at Lys75 by p300, which facilitates chromatin relaxation. This open chromatin state allows recruitment of repair factors for homologous recombination (HR) or non‐homologous end joining (NHEJ). Inhibition of CTPS1 impairs this process, sensitising cells to DNA damage.

Our recent study showed that CTPS1 was highly correlated with radiotherapy resistance in various human cancers, and CTPS1 deficiency also dramatically increased radiotherapy efficacy in cells and xenograft models. The findings indicate the development of CTPS1‐targeted therapies not only as a general means to cancer treatment, but more importantly, to re‐sensitise resistant cancer cells to genotoxic damage. Strategies that develop a specific CTPS1 inhibitor or target CTPS1 phosphorylation, and then combine CTPS1 inhibition with radiotherapy, may be more effective for cancer patients (Figure 1). Clinical trials will be necessary to assess the safety and efficacy of targeting deamidation in human patients, particularly in combination with existing therapeutic strategies.

Interestingly, upon DNA damage, histone H1 is reported to be removed from chromatin, and one of the variants (H1.2) proceeds to degradation, whereas how this deprival is regulated is unknown.^13^ Therefore, deamidation may be one type of enzyme‐motivated PTM to be essential for cellular progressions. Our present study revealed that DNA damage agents stimulate a deamidase CTPS1, to recruit to DSB sites to catalyse H1N76/77 deamidation, which may lead to a structure likely to be degraded. More research is also needed to address whether H1 deamidation is associated with H1 degradation.

## AUTHOR CONTRIBUTIONS

Tingting Feng, Yuan Tian, and Wei‐Guo Zhu wrote the manuscript. Tingting Feng, Mengyan Li, and Chenmiao Hu drew the figure in BioRender.

## CONFLICT OF INTEREST STATEMENT

The authors declare no conflict of interest.

## FUNDING INFORMATION

This work was supported by grants from the Shenzhen Medical Research Fund (B2302010).
